# Supplementary material for: Skin-associated Corynebacterium amycolatum shares cobamides
Source: bioRxiv. 2024 Apr 28:2024.04.28.591522. Preprint. [Version 1] doi: 10.1101/2024.04.28.591522 (PMC11071462; doi:10.1101/2024.04.28.591522)
Supplement: Supplement 1 — Supplemental Figure 1 E. coli metE− and E. coli metE− ΔmetH (initial OD600=0.01) were cultured for 6 h at 37°C, with or without 0.25 ng/mL cyanocobalamin (B12) or 10 μg/mL methionine (Met). Results confirm inability of E. coli metE− ΔmetH to utilize cyanocobalamin for growth. Supplemental Figure 2. Representative cyanocobalamin chromatogram of cell extract from C. amycolatum LK19 WT (250 nM CoCl2). Sample was diluted 50-fold on column to fall within the standard curve. Top panel shows the cyanocobalamin levels in the sample and bottom panel is the internal standard. Cyanocobalamin was quantified in this sample at 14.931 μg/mL. The dotted line represents the mid-point of the chromatogram. Supplemental Figure 3. Cobalt availability regulates C. amycolatum cobamide biosynthesis and sharing with spatially separated cobamide auxotroph E. coli metE−. C. amycolatum WT cell suspension was added to half of a minimal medium plate (0 nM or 250 nM CoCl2) and incubated for 3 days, after which E. coli metE− (blue) was struck out on the adjacent side of the plate. E. coli growth was recorded after 24 h. Growth summary for all conditions are indicated in F). - indicates no growth and +, ++, +++ indicate increasing levels of growth, respectively. (A) C. amycolatum WT lawn on medium with 0 nM CoCl2 and (B) C. amycolatum WT lawn with 100 ng/mL CnCbl spotted on top. (C) C. amycolatum WT lawn on medium with 250 nM CoCl2. 0, 10, and 100 ng/mL Cncbl standards + E. coli metE− on minimal medium plates with (D) 0 nM CoCl2 or (E) 250 nM CoCl2. Supplemental Figure 4 (A) A novel screen was designed to test for biosynthesis of cobamides by C. amycolatum using the indicator strain E. coli metE−. UV mutagenized colonies of C. amycolatum were patched onto minimal medium, followed by spotting of E. coli metE− adjacent to the patches, after which growth of E. coli was assessed after 18 hours. Potential mutants were then validated using a similar assay, with growth of E. coli in response to the potent [file media-1.pdf]

Supplemental Table 1. Plasmids used in this study.		
**Plasmid**	**Description**	**Reference**
pKD4	*bla,* FRT-*kan*-FRT	(27)
pKD46	*bla*, *repA101*(ts), *araBp-gam-bet-exo*, *oriR101*, *araC*, *[tL3]*	(27)

**Supplemental Table 2. Primers used in this study.**		
**Primer Name**	**Primer Sequence (5’ -> 3’)**	**Reference**
MetH_Upstream_Fwd	AGTATGGTTTGTTGAATTTTTATTAAATCTGGGTTGAGCGTGTCGGGAGCGTGTAGGCTGGAGCTGCTTC	This study
MetH_Downstream_Rev	GAGACAGGGCACTCATTGAGCGCCCTGTTTGTTGTAAGGAAAAGTGACAGATGGGAATTAGCCATGGTCC	This study
k1	CAGTCATAGCCGAATAGCCT	(27)
k2	CGGTGCCCTGAATGAACTGC	(27)
kt	CGGCCACAGTCGATGAATCC	(27)
MetH_Outside_Fwd	CCATGACAGCCTCCTTTTTC	This study
MetH_Outside_Rev	GGCATCTTTATTGTGCCTTTTGAC	This study

Supplemental Table 3. Cobamide concentration comparison between indicator assay and mass spectrometry.

		E. coli indicator assay	*Mass spectrometry*
	**Strain**	**Cobamide concentration (μg/g)**	**Cyanocobalamin concentration (μg/g)**
Cell extract	*C. amycolatum* WT (0 nM $CoCl_2$)	0.079 ± 0.003	0.017 ± 0.005
	C. amycolatum WT (250 nM $CoCl_2$)	4.58 ± 1.5	1.17 ± 0.37
	C. amycolatum cob$^-$ (50 nM $CoCl_2$)	0	<LOQ
	C. glucuronolyticum	0.051 ± 0.004	<LOQ

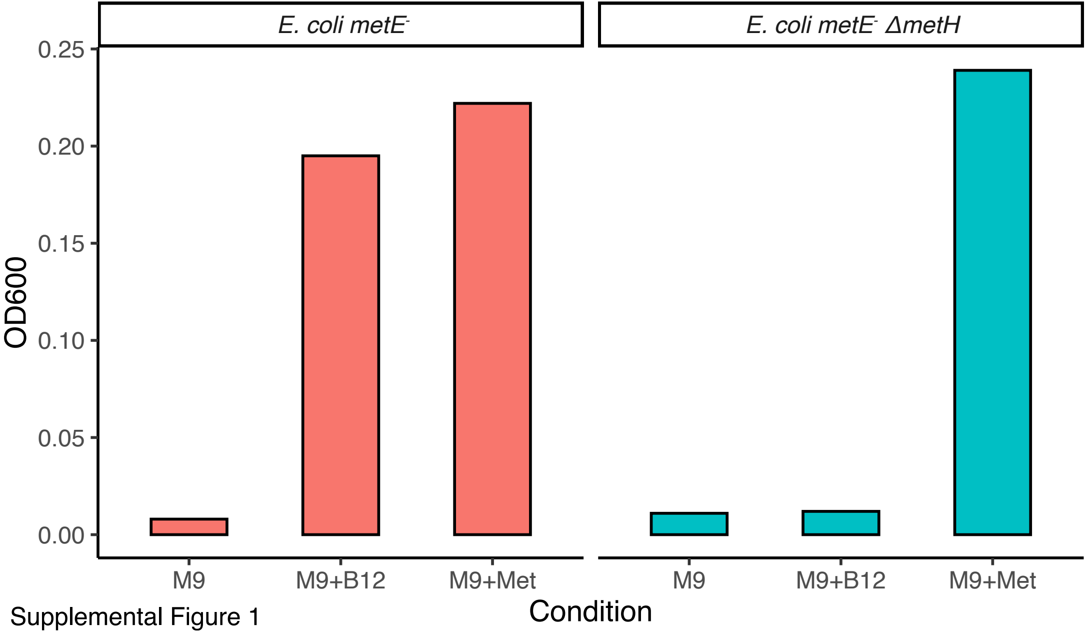

Supplemental Figure 1

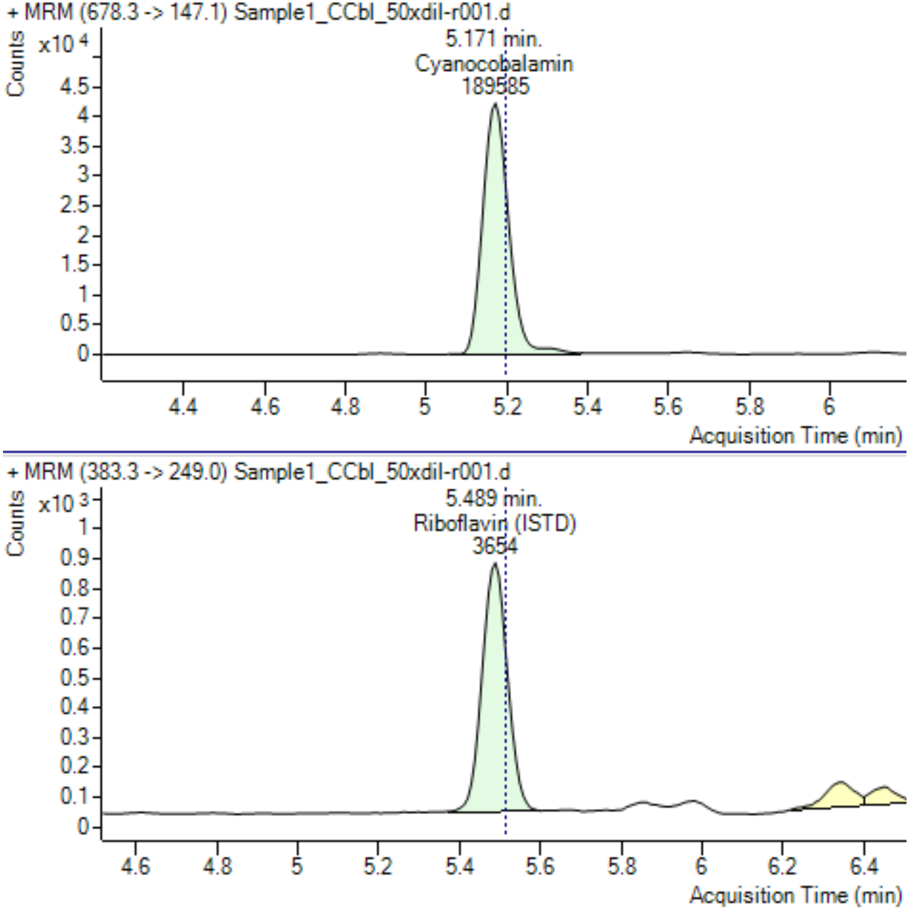

Supplemental Figure 2

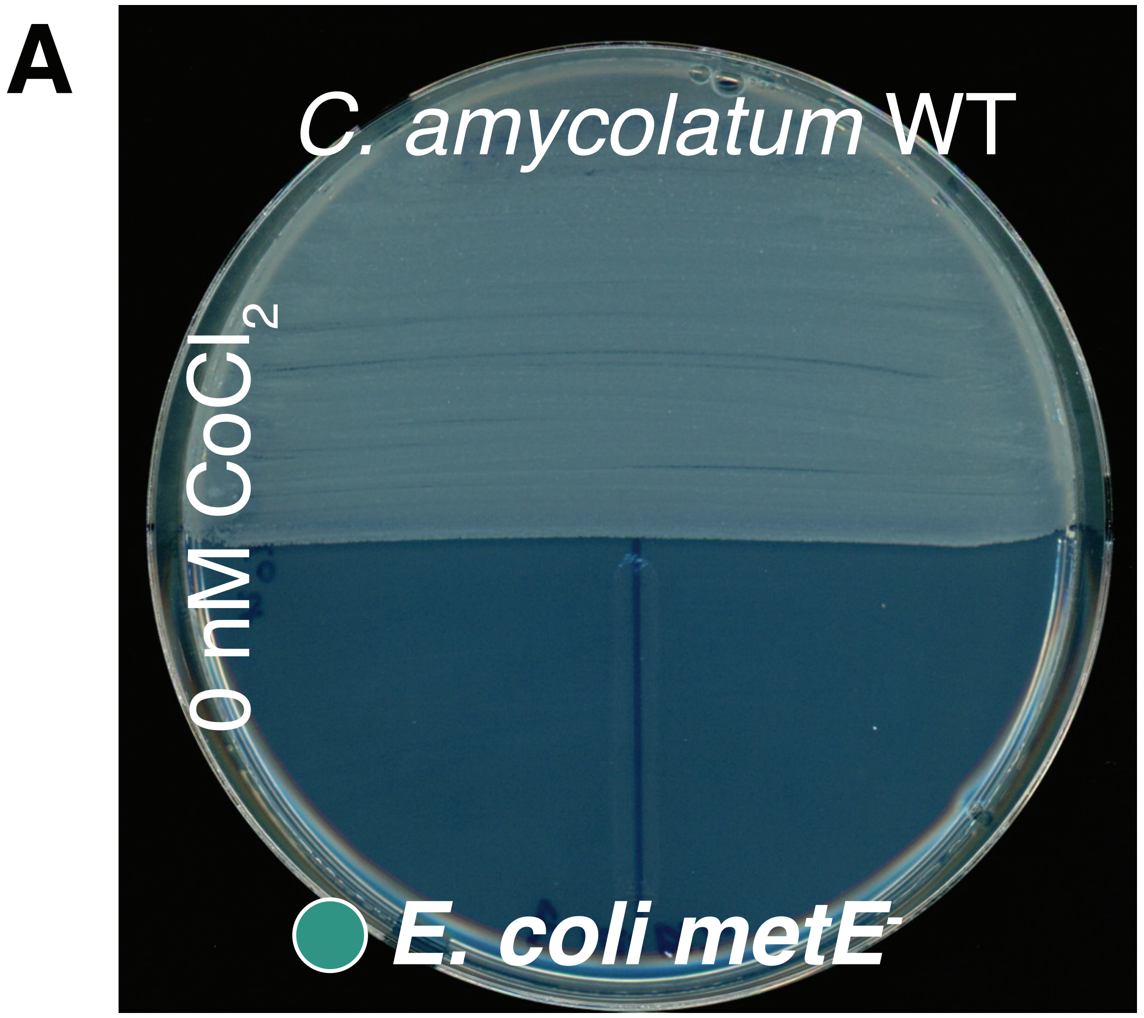

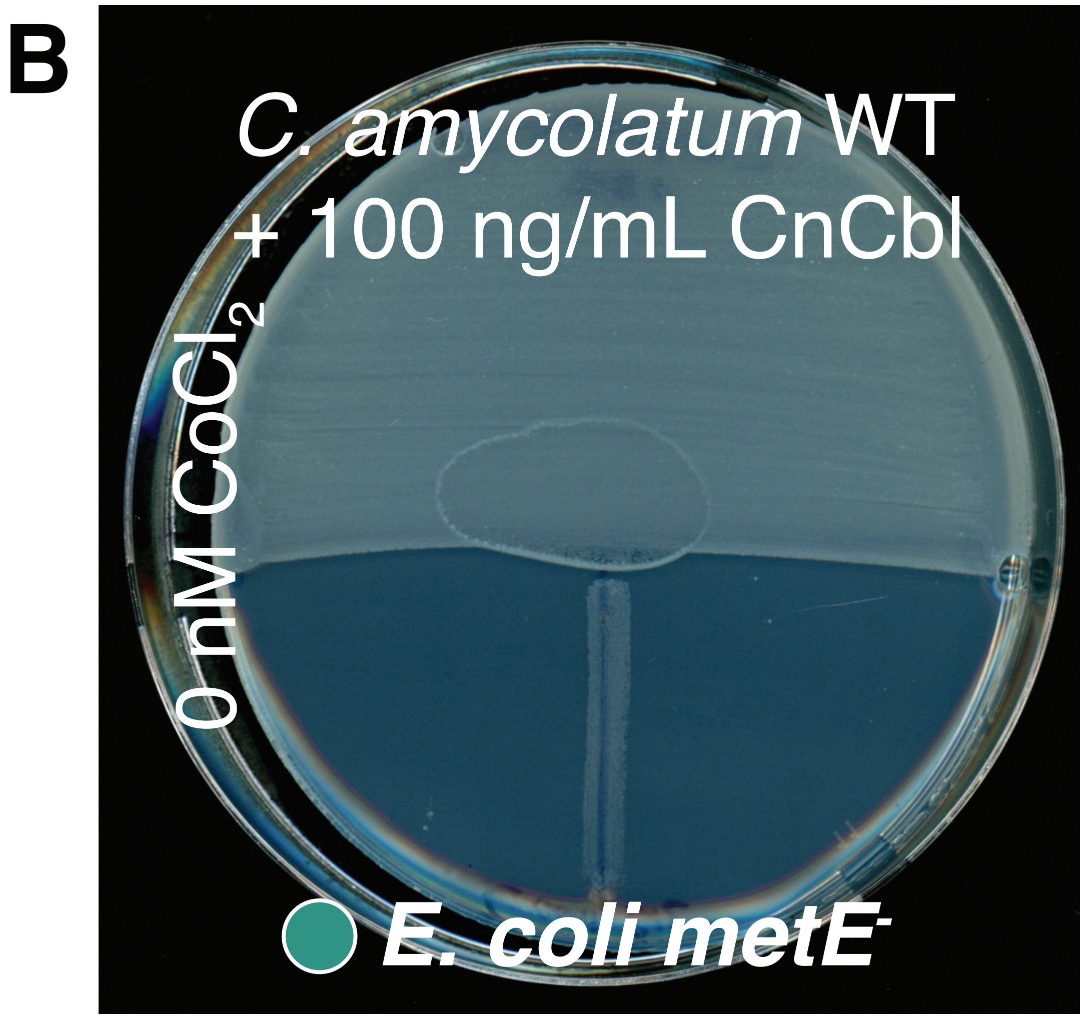

F

Condition	0 nM $CoCl_2$	250 nM $CoCl_2$
C. amycolatum WT	-	++
C. amycolatum WT + 100 ng/mL CnCbl	+++	ND
Control	-	-
10 ng/mL CnCbl	+	+
100 ng/mL CnCbl	+++	+++

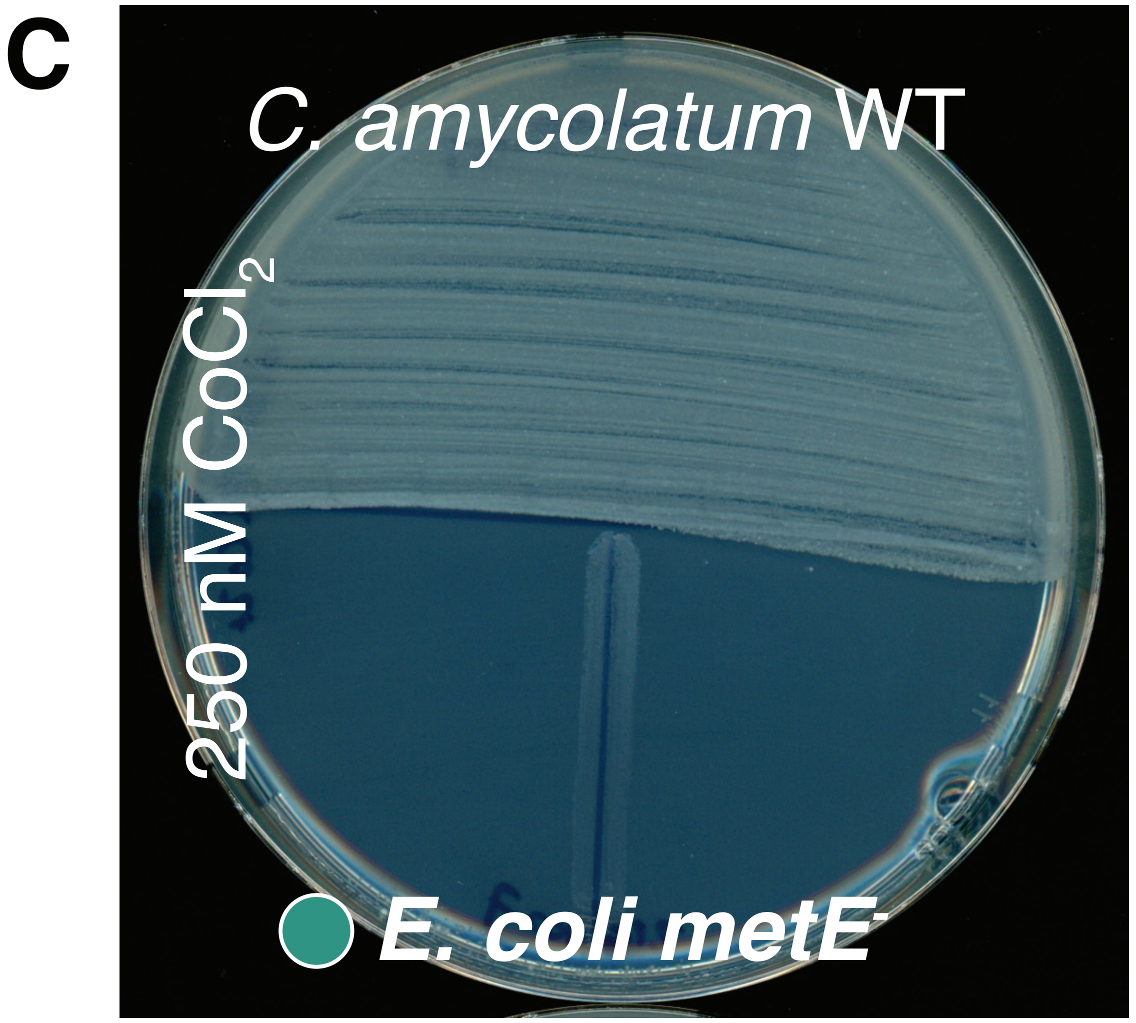

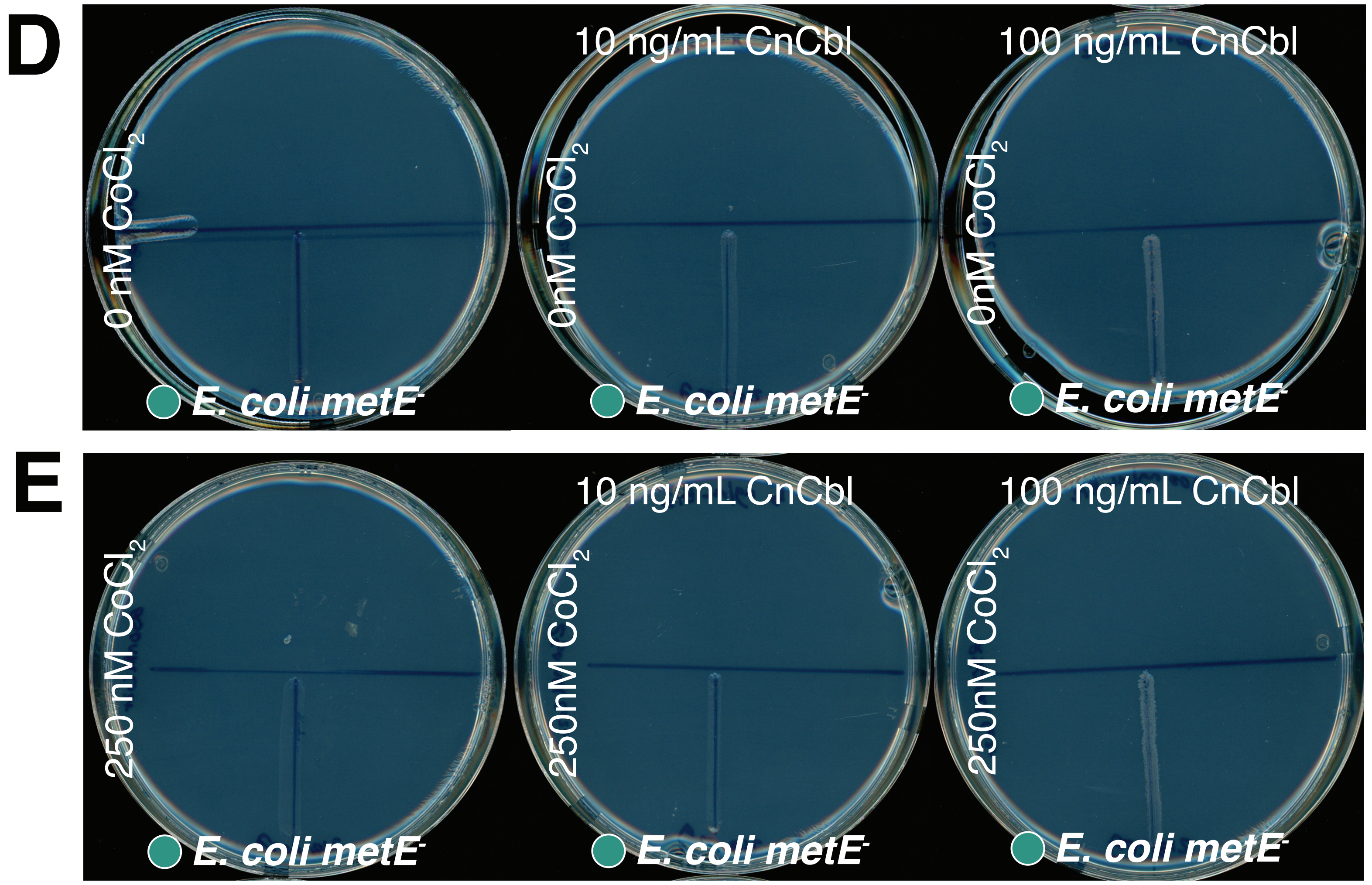

Supplemental Figure 3

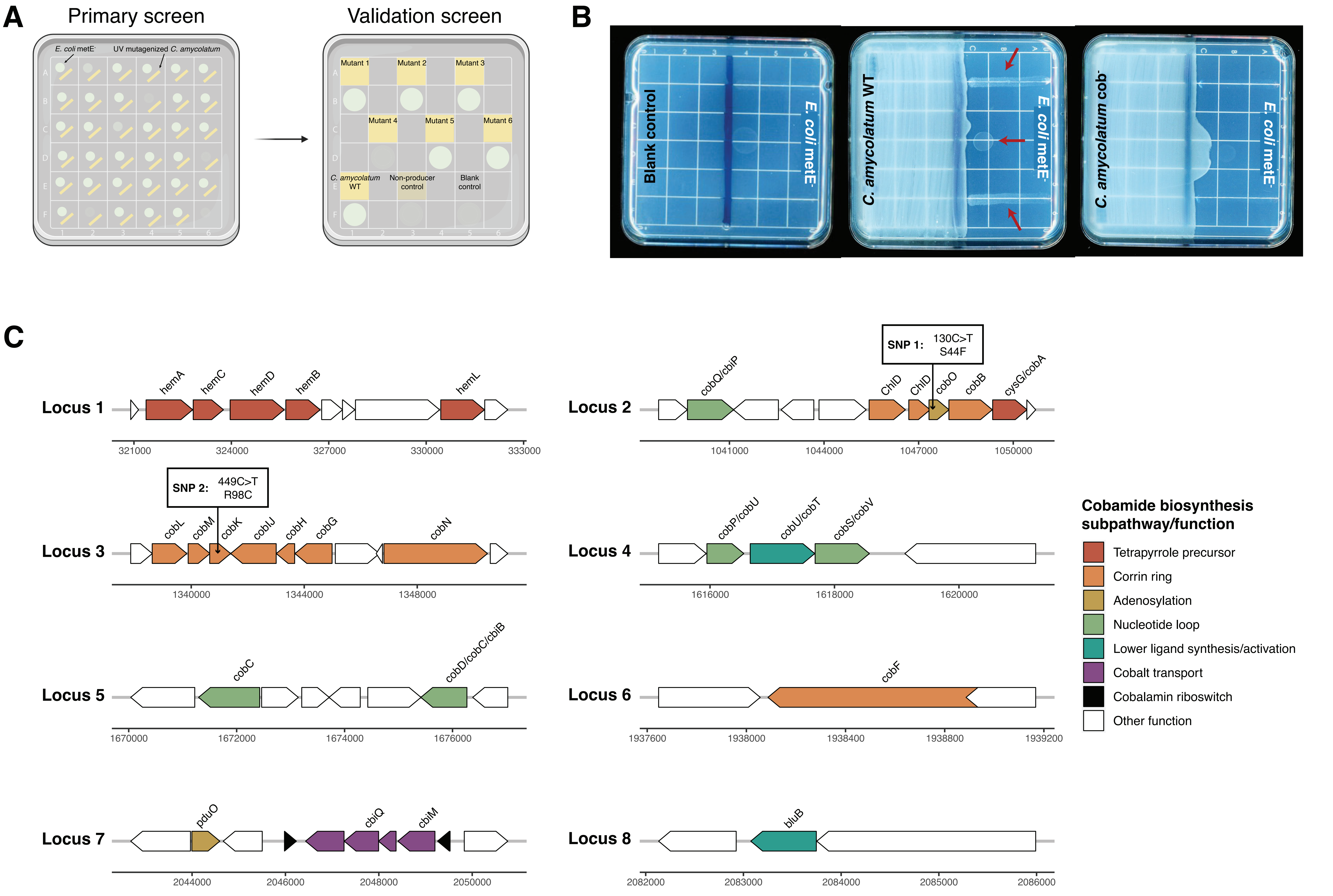

Supplemental Figure 4

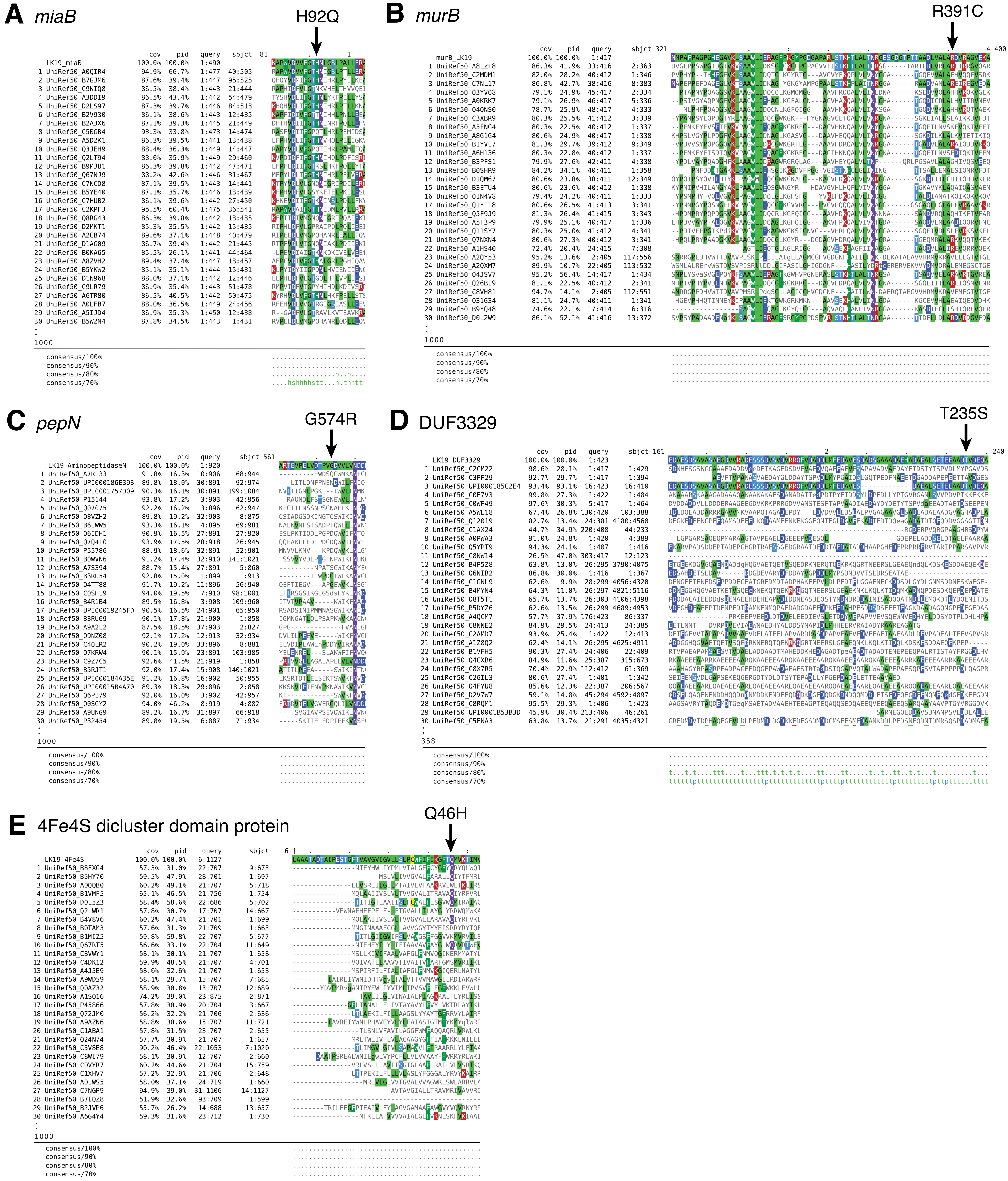

Supplemental Figure 5

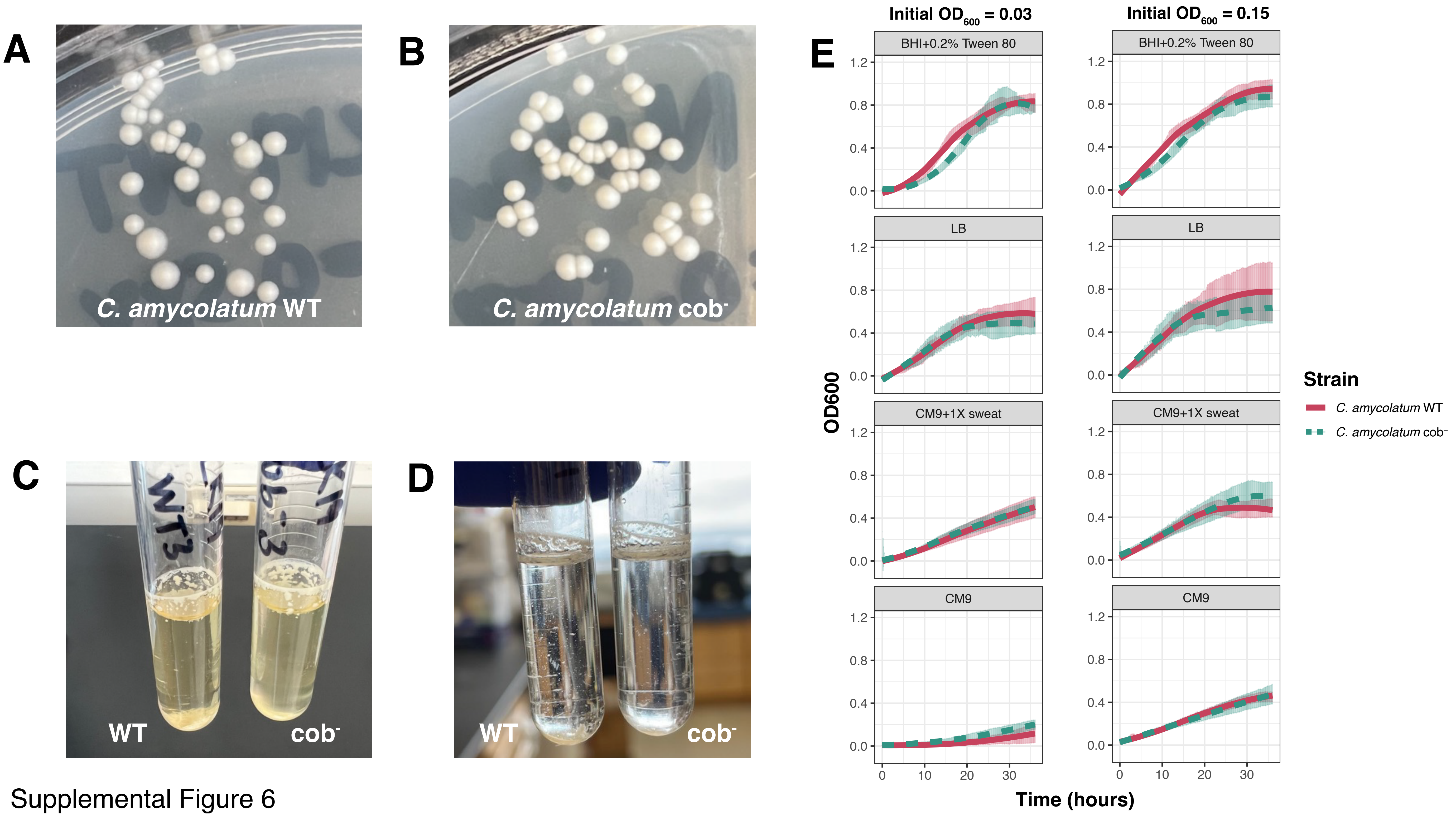

Supplemental Figure 6
